# Association between congenital nasolacrimal duct cyst and bilateral choanal atresia

**DOI:** 10.1590/S1808-86942011000200019

**Published:** 2015-10-19

**Authors:** José Faibes Lubianca Neto, Gabriel Kuhl, Mariana Magnus Smith, Person Antunes de Souza, Leonardo Radünz Vieira

**Affiliations:** 1 MD - Federal University of Rio Grande do Sul - UFRGS (1991); 1a ENT Residency at Hospital de Clínicas de Porto Alegre (1992-1993), MSc (1997) and PhD (2000) in Medicine: Medical Sciences - UFRGS. Fellowship at the Pediatric ENT Division at the Massachusetts Eye & Ear Infirmary, Harvard Medical School, Boston, USA (1997-1998) (Adjunct Professor IV Of the Federal University of Health Sciences - Porto Alegre, Professor at the Graduate Program in Medical Sciences - UFRGS, Director of the Pediatric ENT Division at Hospital da Criança Santo Antônio do Complexo Hospitalar Santa Casa de Porto Alegre; 1b Scientific director of the Associação Gaúcha de Otorrinolaringologia; 1c Chairman of the ENT committee of the Pediatric Otorhinolaryngology Socity of RS and member of the Management Core of the ENT Department of the Brazial Association of Pediatrics; 1d Board Member at the Brazilian Association of Otorhinolaryngology and Neck and Facial Surgery, in the International Relations and Teaching, Residency and Training Departments; 2 MD - University of Coimbra (1973), MD - Universidade de Caxias do Sul (1977) Medical Residency at the Hospital de Clínicas de Porto Alegre (1979) . Assistant Professor III - Federal University of Rio Grande do Sul; Other - Founding Partner of the Brazilian Association of Laryngology and Voice and aide at the Society of Otorhinolaryngology of Rio Grande do Sul; 3 MD - Pontifícia Universidade Católica do Rio Grande do Sul (1994); MD - Universidade Federal do Rio Grande do Sul (2000) and Medical Residency at the Hospital de Clínicas de Porto Alegre (2004) (Effective Member of the Brazilian Association of Otorhinolaryngology and Special Graduate Student at the Hospital de Clínicas de Porto Alegre. Experienced in Medicine, with enphasis in Otorhinolaryngology); 4 MD - Universidade Federal de Pelotas (2006). Resident Physician at Santa Casa de Misericórdia de Porto Alegre; 5 MD-Universidade Federal de Ciências da Saúde de Porto Alegre (2009)

**Keywords:** nasal obstruction, choanal atresia, nasolacrimal duct

## INTRODUCTION

Choanal atresia (CA) is defined as a failure in the development of communication between the nasal cavity and the rhino pharynx, causing complete obstruction of the nasal air flow^1^, ^2^. A congenital cyst of the nasolacrimal duct (CCNLD) happens through an obstruction of the Hasner valve, located in the distal portion of the duct. Despite the uncommon occurrence, such cysts must be included in the differential diagnosis of persistent nasal obstruction at birth^3^, ^4^. The association of both anomalies seems to be extremely rare, and there is only one case reported in the literature^3^.

In the present study there are two cases described of the association between CCNLD and CA. although being a rare finding, the association found in the cases draws our attention to the need to consider other congenital nasal anomalies in individuals with CA.

## CASE REPORT

### CASE 1

**Figure 1:**
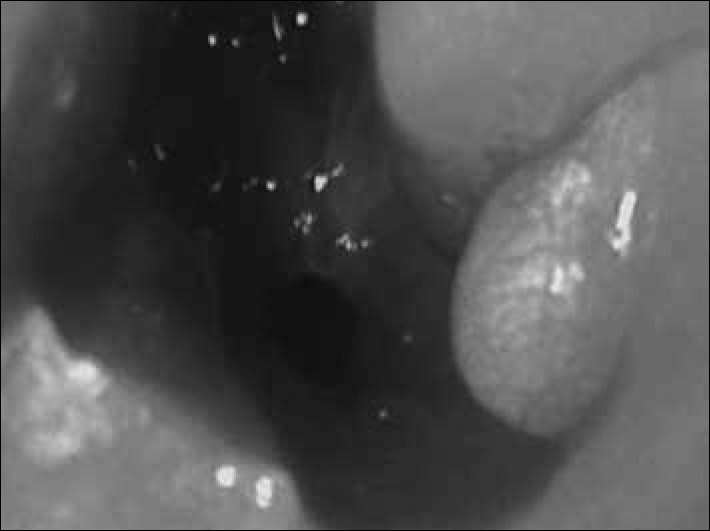
Surgical approach.

Female neonate, at 37 weeks of gestation, presented with progressive respiratory failure immediately after birth. The maneuver of pushing a probe through the nasal cavities was not efficient, leading to the suspicion of complete CA. Since there was a progressive worsening of the patient's respiratory status, we proceeded with orotracheal intubation to stabilize the patient. Facing the initial suspicion of CA, we ordered a skull and face CT scan, which showed bilateral CA, and also a mass in the left-side inferior meatus, as well as dilatation of the ipsilateral lachrymal ducts and sac. With a nasal fiberoptic endoscopy (NFE) done in the operation room we confirmed CA and found a bluish-cystic lesion in the left-side inferior meatus. Such lesion was masupialized, draining a mucoid-looking secretion. Following that, the choanas were bilaterally open through endoscopy (Figure 1). The patient evolved well and was successfully extubated after recovery from anesthesia. The patient was discharged under normal ventilation. After 1 year the patient remained well, without recurrences of the choanal stenosis or that of the cyst.

### CASE 2

Female neonate with respiratory dysfunction immediately after birth, in whom it was also difficult to progress with the aspiration tube in both nasal cavities. She was then intubated and her condition stabilized. Upon NFE done in the ICU, we noticed a bilateral CA and a cystic image in the left nasal cavity. A Facial CT scan confirmed bilateral CA, with a meaningful posterior thickening of the vomer. The patient was also seen by the genetics team, who ruled out other malformations. Two days later the CA was corrected by endoscopic surgery and suffered cyst marsupialization. There were no complications during or after surgery, and the patient developed a normal ventilatory pattern, without intubation. The patient was discharged without symptoms two days after surgery. Today, the patient is 24 months old and the endoscopic exam revealed that both choanas are patent, without cyst recurrence.

## DISCUSSION

Choanal atresia is more common among females^1^, ^2^, which we also found in our sample. They can be uni or bilateral, and 60-70% are unilateral^1^, ^5^. Both described cases were bilateral, as it happened to most of the patients who required early intervention. The incidence is 1 for every 5-7 thousand live births. The diagnosis is ideally established in an urgent basis immediately after birth through NFE, which is not always available. That is why neonatologists usually are the first to raise the hypothesis of CA by noticing the failure in pushing the nasal aspiration tube farther through the nasal cavity into the pharynx.

Paranasal sinuses CT scan and NFE are the gold standard tests^1^, since they set the diagnosis and enable the examiner to identify the type of atresia. Historically, CAs were described as bony in 90% of the cases and membranous in 10%. However, recent studies suggest that the mixed form is the most common (70%), present in the two cases described here. Such differentiation is important when one selects the surgical treatment.

There are basically three surgical techniques for the treatment of CA: transnasal punction (TN), transpalatine correction (TP) and the endonasal endoscopic technique (EE). Today, the EE is preferred for it does not have high recurrence rates such as TN, not so many complications as the TP^1^, ^5^.

Associated congenital anomalies can be found in approximately 50% of the cases, and the CHARGE syndrome (coloboma, heart disease, mental retardation, genital and ear anomalies) is the most frequently described condition. Nasal malformations are not frequently seen with CA. In both cases here reported the patients did not have systemic anomalies; notwithstanding, they both had CCNLD.

CCNLD must be considered among the causes of nasal obstruction in the newborn. A bluish mass in the medial corner of the eye may be associated with dacryocystocele with nasal extension. Nonetheless, the absence of such mass does not rule out the possibility of nasolacrimal duct anomalies causing nasal obstruction^3^. CT scan and NFE are the gold standard tests when managing a neonate with nasal obstruction of unknown cause. Such tests may help identify not only the CCNLD, but also the CA, nasal stenosis and other nasal disorders^4^. CCNLD is a cystic protrusion from the nasolacrimal duct ostium, in the inferior meatus area^3^, ^4^.

Most authors suggest a conservative treatment for asymptomatic cysts (massage and hot pads on the face). However, in nasal obstruction or cyst infection, duct canulation or cyst endoscopic marsupialization must be carried out^3^, ^4^. In the cases hereby reported, we chose marsupialization, having seen that the patients would be submitted to surgery anyway to open the choanas.

After an extensive literature review using the Pubmed database, we found only one case associating CCNLD and bilateral CA. Such study presented a female patient with numerous malformations (CHARGE syndrome)^3^. CT and endoscopic aspects were similar to those from the patients described in the present study. Nonetheless, this patient died seven days after birth because of severe malformations.

In conclusion, it is important to notice the association between CA and CCNLD. Although being a rare case, the association found draws our attention to the need to look for other nasal congenital anomalies in individuals with CA.
